# Water and sediment pollution of intensively used surface waters during a drought period — a case study in Central Northern Namibia

**DOI:** 10.1007/s10661-023-11505-1

**Published:** 2023-07-06

**Authors:** Leona Faulstich, Robert Arendt, Christian Reinhardt-Imjela, Achim Schulte, Joachim Lengricht, Petrina Johannes

**Affiliations:** 1 Department of Earth Sciences, Freie Universität Berlin, 12249 Berlin, Germany; 2 Civil and Environmental Engineering, University of Namibia, 3624 Ongwediva, Namibia

**Keywords:** Water quality, Pollution, Sediment, Aluminum, Iishana system, Namibia

## Abstract

Semiarid regions are often affected by water scarcity and poor water quality. Seasonal changes in precipitation and drought events increase the pressure of use on water bodies and their pollution. In Central Northern Namibia, a high seasonal intra- and inter-annual variability of precipitation caused a 5-year lasting drought period. In the semiarid region, ephemeral channels and water pans represent the main water source, besides the institutionalized water supply. No systematic analysis of its quality has been conducted so far. The states of the surface waters at the end of the dry season in 2017 and the end of the rainy seasons in 2018 and 2019 were characterized by the analysis of physical–chemical parameters, focusing on usability. The first results show coarse contamination of the waters, which results in high turbidity values. Salt concentrations, such as Ca^2+^ and Na^+^, greatly increased due to evaporation. Al is present in high concentrations in solid and liquid phases, which indicates direct anthropogenic pollution. Spatial differences are evident in the study area and based on the precipitation gradient, land use, and population density. The waters cannot be used as drinking water without prior treatment.

**Supplementary Information:**

The online version contains supplementary material available at 10.1007/s10661-023-11505-1.

## Introduction

Occurring droughts have a lasting effect on the quality of water bodies in arid and semiarid regions (Li et al., 2018a; Mayer et al., 2010; Olds et al., 2011). Long retention times, low water flow, and reduced flushing during dry periods (Caruso, 2002; Flanagan et al., 2009) result in high turbidity values and salt accumulation (Yan et al., 1996). Evaporation increases nutrient and pollution concentrations (Valcarcel Rojas et al., 2020). In the Iishana system in the west of the Cuvelai Basin (CB) in Central Northern Namibia, drought events frequently occur, last between 2015 and 2019 (Shikangalah, 2020). The semiarid climate of the region is characterized by high evaporation rates and a strong seasonal variability of precipitation with distinct dry and rainy seasons (Masih et al., 2014; Mufeti et al., 2013; NEWFIU, 2015; SADC, 2013). In the rainy season, precipitation is concentrated on a few storm events per month (Kluge et al., 2008; Reason & Smart, 2015). The ephemeral drainage system with its depressions, called Iishana (singular Oshana, see Arendt et al., 2021 for details), provides water for the rural population, though nearly no data exist on the hydrological system and the quality of surface waters (Christelis & Struckmeier, 2011). During the drought period, water levels in these water bodies decreased continuously due to evaporation, anthropogenic use, and endangered water supply. In addition, the CB and in particular the Iishana system are among the most densely populated areas in southern Africa which further increase the pressure on water resources. The intensive use of water bodies results in various potential sources of chemical pollution. Until now, there are no studies that focus on the conditions of the surface waters in the Iishana system, even though it is an important water resource.

In the neighboring Okavango Delta, heavy and light metals were found in surface waters, partially at trace-level concentrations (Dauteuil et al., 2021; Mmualefe & Torto, 2011). A concentration effect due to high evaporation was observed. In Nigeria, heavy metals in surface waters caused health risks (Tenebe et al., 2019). Increased light and heavy metal concentrations in the water column cause severe health problems with consumption (Chowdhury et al., 2016). With a decreasing pH the toxicity of metals increases (Campbel & Stokes, 1985). In sediments, aluminum for example reacts in an acid environment (Rengel, 2004) with a toxic effect on plants and their growth, a major problem in agriculture (DeForest et al., 2018). The toxicity and solubility of aluminum increase in acid and strong alkaline environments, with pH below 6 and above 8 (Wilson, 2012).

Previous studies in the area mainly focused on the quality and isotopes of groundwater (Hamutoko et al., 2016, 2018, 2019; Lindenmaier et al., 2012) and hand-dug wells accessing shallow groundwater (Wanke et al., 2014). In these wells, mineralization and turbidity are often high and recharge is low, which decreases the potential use as drinking water (Wanke et al., 2014). All these studies reveal a great need for additional water sources, but none of them examine the Iishana. The Iishana already cover part of the water demand for households, subsistence agriculture, and livestock farming (Kluge et al., 2008), even if the consequences are unknown. Some studies already point out this grievance and the lack of data (Klintenberg et al., 2007; Liehr et al., 2017).

This study aims to improve the knowledge of the water quality and usability of the Iishana pans by evaluating the condition of water bodies during a drought period based on nutrients, pollutants, and sediment loads, to figure out possible pollution sources. Is the water of the Iishana usable for human consumption without health risks? Are there any spatial variations in the study area? Without reliable data on the surface waters, structured water use measures cannot be established. Understanding the status of surface waters is also necessary to better understand groundwater reserves. The selection of the measurement parameters is based on the requirements of the World Health Organization (WHO, 2017). Samples were taken at the end of the dry season in 2017 and at the end of the rainy season in 2018 and 2019. The results are compared with standards and limit values of the Namibian Water Act 54 (DWAF, 1990) and international regulations for drinking water quality (Guideline for Drinking-water Quality of the World Health Organization; WHO, 2017).

## Material and methods

### Study area

**Fig. 1:**
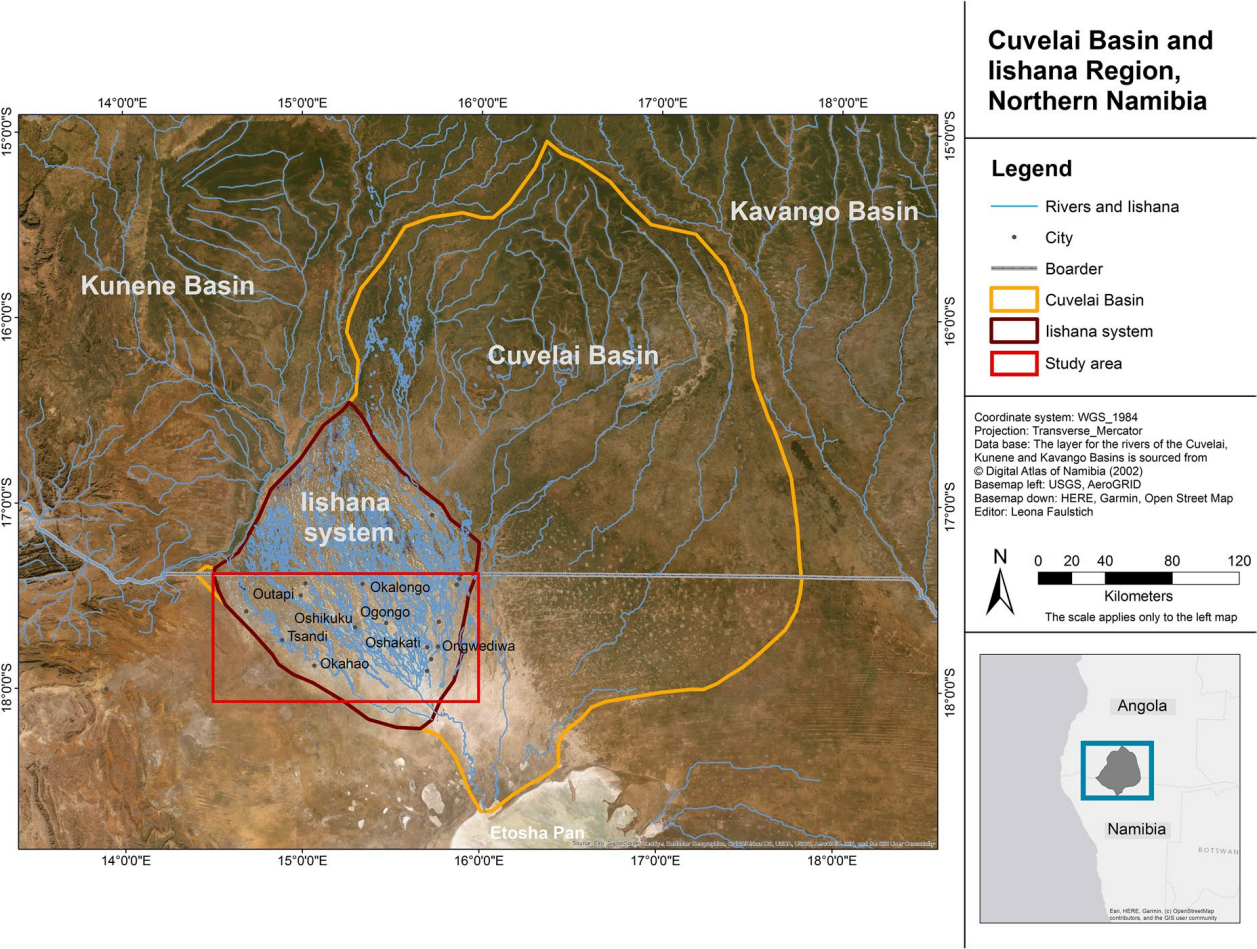
The Cuvelai Basin with the Iishana system, neighboring river systems (Kunene and Kavango catchments) and the study area (red box)

The Iishana system, as part of the CB, is a transboundary drainage system that leads from the planalto midlands in Angola through flat areas in northern Namibia and discharges into Lake Oponono. The study area lies in the Iishana system with an area of 18,370 km^2^, 8,726 km^2^ on the Angolan site, and 9644 km^2^ on the Namibian territory (Fig. 1). The area is located at an elevation between 1100 and 1200 m.a.s.l. and has a mean slope of 1‰ (Calunga et al., 2015; Mendelsohn et al., 2013; Persendt & Gomez, 2016), which results in a very slow runoff and increased salinization processes. Saline surface sediments are dominated by fluvial sands, calcretes, and calk crusts that cause low infiltration rates and strong surface runoff (Eitel, 1994; Goudie & Viles, 2015; Lindenmaier et al., 2014).

The shallow basin enhances salinization processes in surface water, sediments, and groundwater and is a challenge for the utilization of natural resources. Most of the aquifers in the CB are located at a depth between 250 and 350 m, which impedes abstraction and distribution, so these deep aquifers can only be used to a limited extent, also because the groundwater is strongly over salted (Himmelsbach et al., 2018; Mendelsohn et al., 2013; Seely et al., 2003). Several shallower perched aquifers are in a depth between 10 and 40 m, which facilitates extraction; however, salinity and pollutant concentrations are enormous (Bäumle & Himmelsbach, 2018; Christelis & Struckmeier, 2011).

In the northern part of the Iishana system, the perennial rivers Cuvelai and Mui provide water for the Angolan part of the basin (Mendelsohn et al., 2013). On the Namibian side, local, episodic precipitation, and surface runoff are the only water sources. This ephemeral and endorheic system (Endorheic basins are systems without an outflow into the ocean, but into an inland body of water, such as a lake, or in this case the Etosha Pan.) consists of channels and embedded natural depressions (Arendt et al., 2021; Miller et al., 2010; Seely et al., 2003). With sufficient rainfall or during large-scale flood events, the depressions fill up and connect to form a large drainage system of thousands of small, branched channels that end in the Etosha Pan (Awadallah & Tabet, 2015; Curtis et al., 1998; Hiyama et al., 2014; Hooli, 2015; Lindenmaier et al., 2014; Seely et al., 2003; Shifidi, 2016).

During the rainy season from October to April, rainfall varies between 350 and 550 mm/a (Mendelsohn et al., 2003 & 2013). The potential evaporation ranges from 2200 to 3500 mm/a (Mendelsohn et al., 2013), which is six times higher than the total rainfall and impedes surface water storage. The decreasing precipitation gradient from east to west affects different small-scale conditions concerning water availability (Mendelsohn et al., 2003). Due to data from the National Weather Service of the National Oceanic and Atmospheric Administration (NOAA) since 2013, the observed precipitation was below average (NOAA, 2020), except for the rainy season in 2015/2016.

**Fig. 2:**
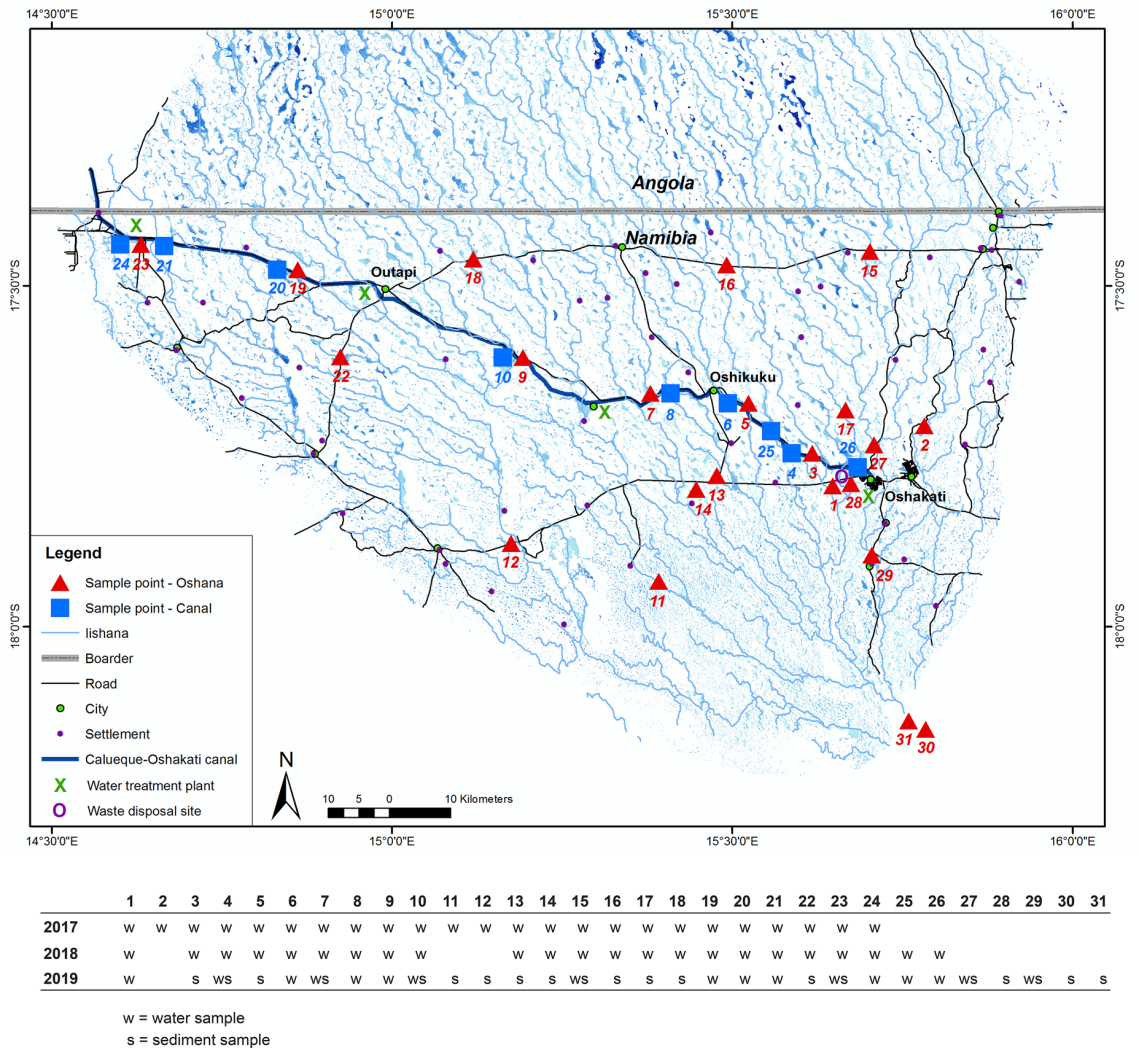
Map of the study area and sample points at the Iishana and the Calueque-Oshakati canal

This complex hydrological system provides water for nearly half of the Namibian population (NSA, 2013) and is under big pressure to meet all supply demands. The main artificial source of water is the 154 km open Calueque-Oshakati canal (COC) that was built as part of the Namibia Water Master Plan in 1974 and transports water from the Kunene Dam by Calueque in Angola over the Olushandja Reservoir in Namibia to Oshakati (Shuuya & Hoko, 2014). In the course of the COC, four water treatment plants were built close to urban areas: Olushandjy, Ombalantu, Ogongo, and Oshakati (see Fig. 2). These plants treat and supply water for the towns and villages of the region. Communities or households that cannot afford charges for treated water use the available surface water from the Iishana or hand-dug wells (Liehr et al., 2017; Shifidi, 2016). In particular, during drought periods, the surface water is usually used for cleaning, irrigation, and livestock. Tap water is rather used as drinking water, for cooking, and washing (Luetkemeier & Liehr, 2015). In extreme situations of water scarcity, people use Iishana water as drinking water. The high population density increases the pressure on water resources. Recent population projections assume a continuous increase in the future. With a growing rate of 1.4% per year (NSA, 2013), the population in the western CB is projected to be 27% higher in 2041 than in 2019 (NSA, 2014). There will be a strain on the public supply system, and the already heavily used and stressed surface waters will be subject to even greater pressure.

### Sampling sites

To analyze the quality of surface waters in the Namibian part of the Iishana system and to identify the influencing factors, an evenly distributed measurement grid that would cover the entire investigation area, considering the precipitation gradient, was applied. Sampling sites at the Calueque-Oshakati canal and the Iishana were chosen to compare water sources. Sample points were selected based on areas still containing water at the end of the dry season in 2017 and resampled at the end of the following rainy seasons in 2018 and 2019. Due to the accessibility of potential sites, only those Iishana were selected, which are located within a maximum distance of 300 m from a road. During the campaigns in 2018 and 2019, many of the previously selected Iishana were no longer filled with water. Only dark sediments, partially not even wet depressions, were left.

Furthermore, the tap water in Oshakati and Ruacana was analyzed to compare untreated surface water with treated water from the supply system, considering that ailing pipes can influence the water quality and do not necessarily correspond to the quality of the water when it is fed into the network by the waterworks. During a rainfall event in March 2018, it was possible to collect rainwater for comparative analysis. A table with applied methods, corresponding test protocols, parameters, and accuracies can be found in supplementary data 1.

### Water samples

The parameters temperature, pH value, redox potential (Eh), oxygen content and saturation, electrical conductivity (EC), turbidity, chlorophyll-α, and cyanobacteria were measured in situ with the YSI-multiparameter probe 6600 V2-4 (Xylem Analytics, Germany). Three water samples were taken from the surface water at a depth of about 0.15 m in a PE beaker according to the DIN protocol 5667–3:2013–03 (DIN, 2013). The samples were taken in PE bottles (0.1 l; VWR, Germany). One sample was filtrated with a syringe filter holder (0.45 µm; Hach Lange GmbH, Germany), acidified with 0.1 ml of nitric acid (HNO_3_; fuming 100%, Carl Roth GmbH + Co. KG, Germany), and cooled for the transport. One unfiltered sample was taken for the analyses of suspended solids. The last sample was transported in a 0.1 l PE bottle to the field laboratory at the UNAM, Campus Ongwediva, for “on-site” analyses. On the same day, the samples were centrifugated (EBA 280, Hettich GmbH & Co. KG, Germany) for 20 min at 6000 min^−1^ to facilitate the following filtration with syringe filter holders (0.45 µm; Hach Lange GmbH, Germany). Afterward, the ion concentrations were measured with the portable HACH DR 1900 VIS spectrophotometer (Hach Lange GmbH, Germany) and a cuvette system. Such portable spectrophotometers were previously used in several studies to analyze water samples in the field (Marlow et al., 2000; Pandey et al., 2019; Yuan et al., 2013; Zhang et al., 2019). Following anions and sum parameters were analyzed: chloride (Cl^−^), fluoride (F^−^), ammonium (NH_4_^+^), nitrate (NO_3_^−^), nitrite (NO_2_^−^), phosphate and orthophosphate (PO_4_^3−^), sulfate (SO_4_^2−^), chemical oxygen demand (COD), total nitrogen bound (TNb), total carbon (TC), total inorganic carbon (TIC), and total organic carbon (TOC).

At the laboratory at Freie Universitaet Berlin, Germany, the acidified samples were centrifuged with a Multifuge 4KR Heraeus Centrifuge (Thermo Fisher Scientific, USA) for 15 min at 10,000 min^−1^. The liquid was pipetted and filtrated with a 0.45-µm membrane filter (cellulose acetate; VWR, Germany) to exclude all suspended solids. After the preparation, the following cations were analyzed with the ICP-OES 2000 (PerkinElmer, USA) according to the DIN protocol DIN EN ISO 11885:2009 (DIN, 2009a): aluminum (Al), arsenic (As), cadmium (Cd), calcium (Ca^2+^), chrome (Cr), cobalt (Co), copper (Cu), iron (Fe^2+^), lead (Pb), magnesium (Mg^2+^), manganese (Mn), nickel (Ni), potassium (K^+^), sodium (Na^+^), strontium (Sr), and zinc (Zn).

The unfiltered samples were centrifuged for 15 min at 10,000 min^−1^ (Multifuge 4KR Heraeus Centrifuge; Thermo Fisher Scientific, USA) and filtered (0.45 µm membrane cellulose acetate filter; VWR, Germany). In total nine Iishana left enough suspended solids (> 0.5 g) for analysis. The solid matter was dissolved with an Aqua Regia digestion (according to DIN EN 16174:2012–11; DIN, 2012a). The new solutions were analyzed with the ICP-OES 2000 for the same ions as the water samples.

### Sediment samples

The sediment samples were taken from the bottom of the dry Iishana, and from the Iishana, which were still filled with water, from the water’s edge. The first centimeters were discarded to exclude aeolian sediments. The sediments were taken with a wooden spoon and transported in plastic bags. In the canal, the sediments were collected with a plastic beaker on an extension rod.

In the laboratory, the samples were homogenized in evaporation dishes and dried for 24 h at 45 °C. The sieving was done with plastic sieves with a diameter of 150 mm and a mesh width of 2.0 mm and 1.0 mm (Test Sieves; VWR, Germany). The rest of the sample was wet sieved according to the DIN protocol 66,165–2:2016–08 (DIN, 2016) through a 0.063-mm sieve (Test Sieve, VWR, Germany; diameter of 150 mm). The grain size distribution was performed by using a Beckman Coulter LS 13 320 laser diffractometer (Beckman Coulter Life Sciences, USA) according to the DIN protocols (DIN, 2009b; DIN, 2018). For determination of the pH value 5 ml of the sample was suspended in a 0.01-mol/l CaCl_2_ solution (≥ 99%, p.a., ACS, Carl Roth GmbH + Co. KG, Germany; DIN, 2012b). For the EC measurement, deionized water was used (DIN, 1993). Total carbon (TC) was determined using a LECO TruSpec Elemental Determinator (LECO, USA). Total inorganic carbon (TIC) was measured using a Carmhograph C16 (Wösthoff; Germany), and total organic carbon (TOC) was calculated. The different carbon contents were determined according to the DIN protocol DIN EN 15936:2012–11 (DIN, 2012c). Parts of the fractions < 0.063 mm and 0.063–1.0 mm were dried at 50 °C for 24 h and digested with Aqua Regia (DIN, 2012a) for analysis with the ICP-OES (DIN, 2009a).

### Water guidelines

Critical values for the quality of water for domestic use and tap water are not specifically established in Namibia. The only Namibian directive for the quality of different types of water is the Water Act 54 of 1956 (DWAF, 1990). It was adopted from South Africa in the 1950s and was updated in 1990 by the Namibian government. The Water Act 54 applies to drinking water and water for domestic use. There are four categories for water analysis that should divide the waters into different groups: (i) group A: water with excellent quality; (ii) group B: water with acceptable quality; (iii) group C: water with low health risk; and (iiii) group D: water with a high health risk or water unsuitable for human consumption (DWAF, 1990).

Considering the results and the classification in an international context, the critical values for drinking water from the World Health Organization (WHO, 2017) are also used. The WHO defines guideline values for parameters, which are harmful to human health (in particular metals like copper, nickel, chrome, lead, cadmium, and arsenic). For other parameters, like ammonium, chloride, iron, manganese, nitrate, potassium, sodium, sulfate, and zinc, no guideline values have been established yet. Usually, these parameters occur in drinking water at concentrations well below those of health concern. Since the water of the Iishana is used as drinking water (Neliwa & Kalumbu, 2019; Sturm et al., 2009), a combination of these two guidelines guarantees an adequate assessment of local surface waters.

### Data analysis

The data analysis was performed by using the scripting language R (Version: 4.2.0) (R Core Team, 2019), in an RStudio environment (Version: 2022–04-22 ucrt) with several packages in Rstudio: “compositions,” “psych,” “car,” “dplyr,” “ggplot2,” “pgirmess,” and “PMCMRplus.” Compositional data, as measured concentrations, are non-symmetrical distributed. Since the numerical space is positive, it is necessary to use the geometric mean instead of the arithmetic mean (Aitchison, 1994; Greenacre, 2021). To perform the statistical tests adequately, the data were log-transformed (centered log-ratio clr transformation) before testing. After performing the tests, the data were retransformed. Each parameter was tested for normal distribution in each campaign using the Shapiro–Wilk test. A Levene test was performed to check the differences in variances. The not normal distributed parameters with unequal variances were tested with a Friedman test to gain knowledge of the differences between sampling sites (Iishana and COC) as well as between dry and wet seasons. As a post hoc test, the Wilcoxon test was carried out. The sample sizes of suspended solids of the COC and the Iishana in 2019 were too small for statistical analysis. The data of suspended solids in 2017 and 2018 are paired and normally distributed; therefore, the paired *T*-test was performed. Differences between the Iishana and the COC were tested with the Mann–Whitney *U*-test (95% confidence interval), because of unrelated and not normally distributed data. The statistical analysis results were defined as significant with a *p*-value < 0.05.

## Results

### Hydrochemistry and water quality

At the end of the dry season in 2017, the sampled Iishana were filled with water; after the wet season in 2018, three Iishana were dried up; and at the end of the wet season in 2019, eleven more. The rainy seasons in 2018 and 2019 were not strong enough to fill every Iishana with sufficient water. Water levels varied between 0.2 and > 0.7 m (a detailed table can be found in the supplementary data 2). Ranges, means, and standard deviations of physicochemical parameters and major ion concentrations are presented in Table 1. Several parameters, like carbon, oxygen, and heavy metal concentrations, are not discussed in the text, but can be found in the supplementary data 3.

**Table 1:** Range, mean, and standard deviation (sd) of the hydrochemical composition of Iishana, Calueque-Oshakati canal, tap water, and precipitation. The temperature in degree Celsius, EC in microsiemens per centimeter, turbidity in NTU (nephelometric turbidity unit), and major ions in milligrams per liter. Limit values of the guidelines Namibian Water Act 54 from 1956 and Guidelines for Drinking-water Quality of the WHO from 2017

	**Iishana**						**Calueque-Oshakati canal**					
	***n* = 19**						***n* = 9**					
	**Range**			**Mean ± sd**			**Range**			**Mean ± sd**		
	**2017**	**2018**	**2019**	**2017**	**2018**	**2019**	**2017**	**2018**	**2019**	**2017**	**2018**	**2019**
	**Dry**	**Wet**	**Wet**	**Dry**	**Wet**	**Wet**	**Dry**	**Wet**	**Wet**	**Dry**	**Wet**	**Wet**
**Temp**	20.1–31.2	23.3–33.2	22.4–32.1	25.0 ± 3.3	28.8 ± 2.2	27.2 ± 2.8	21.1–28.0	25.5–29.3	23.3–31.7	24.8 ± 2.1	26.6 ± 1.2	26.7 ± 2.9
**pH**	7.3–9.4	6.3–8.6	7.0–9.2	8.2 ± 0.6	7.4 ± 0.8	8.1 ± 0.7	7.3–8.9	6.5–8.1	6.4–7.7	8.0 ± 0.5	7.3 ± 0.4	7.0 ± 0.5
**EC**	80–2230	90–2020	76–2935	911.2 ± 669.9	614.7 ± 655.4	724.9 ± 229.3	74–99	43–98	64–109	82.3 ± 7.8	71.9 ± 17.7	75.0 ± 13.2
**Turbidity**	17.5–1471.6	47.9–1422.3	16.1–1615.4	631.5 ± 551.6	722.2 ± 545.9	477.6 ± 496.7	6.5–17.6	64.3–317.6	53.5–290.5	12.7 ± 3.6	164.8 ± 96.2	196.5 ± 83.2
**Cl**^**−**^	3.9–554.0	9.28–678.0	3.9–342.0	206.9 ± 179.0	170 ± 209.1	71.4 ± 105.4	1.3–2.8	3.3–18.1	1.1–3.9	1.91 ± 0.5	8.8 ± 5.4	2.2 ± 0.7
**SO**_**4**_^**2−**^	72.5–707.1	48.7–1224.0	47.0–309.2	252 ± 171.1	298 ± 334.3	126.1 ± 78.9	< 1.0	47.3–96.7	35.5–68.6	-	73.1 ± 19.4	50.1 ± 14.6
**NO**_**3**_^**−**^	0.1–3.5	1.6–24.1	0.2–3.8	1.6 ± 1.1	8.5 ± 6.9	1.0 ± 1.1	0.02–0.24	1.8–6.0	0.3–0.6	0.2 ± 0.1	3.1 ± 1.3	0.4 ± 0.1
**NO**_**2**_^**−**^	0.03–0.5	0.05–7.9	0.03–0.1	0.2 ± 0.2	2.0 ± 2.6	0.07 ± 0.05	0.01–0.02	0.2–0.9	< 0.01	0.020 ± 0.001	0.5 ± 0.3	-
**NH**_**4**_	0.06–2.0	0.08–12.4	0.02–0.2	0.5 ± 0.5	2.6 ± 3.5	0.07 ± 0.1	0.02–0.07	0.07–8.58	0.02–0.06	0.04 ± 0.02	1.8 ± 3.1	0.03 ± 0.01
**PO**_**4**_^**3−**^	0.1 -1.5	0.1–37.1	0.05–4.5	0.6 ± 0.4	7.5 ± 10.8	0.9 ± 1.4	< 0.05–0.3	0.4–1.8	0.1–0.3	-	0.9 ± 0.5	0.32 ± 0.01
**F**^**−**^	0.3–1.0	0.1–0.3	0.2–2.8	0.6 ± 0.3	0.2 ± 0.1	0.8 ± 0.9	0.1–0.2	< 0.1	0.1–0.2	0.15 ± 0.04	-	0.12 ± 0.01
**K**^**+**^	1.9–17.5	3.0–26.9	0.3–9.6	8.6 ± 4.6	9.7 ± 7.2	5.6 ± 3.6	1.5–17.1	1.7–4.4	0.1–0.5	4.1 ± 5.3	3.0 ± 0.8	0.3 ± 0.2
**Na**^**+**^	5.8–397.0	10.9–429.0	2.1–196.5	132.1 ± 112.3	109.2 ± 126.9	67.4 ± 65.4	5.1–281.8	4.5–15.4	3.7–6.8	45.8 ± 96.4	8.8 ± 3.7	4.6 ± 0.9
**Mg**^**2+**^	2.0–17.7	3.6–27.2	2.3–17.5	8.6 ± 4.3	13.1 ± 6.9	6.8 ± 4.2	1.9–28.7	1.8–8.2	1.6–2.1	5.9 ± 9.3	4.7 ± 2.2	1.8 ± 0.1
**Ca**^**2+**^	6.0–37.7	5.8–51.4	3.8–26.9	17.2 ± 8.4	28.7 ± 9.6	15.8 ± 6.6	5.7–42.8	6.2–26.4	4.9–7.0	11.7 ± 12.7	16.1 ± 7.7	5.9 ± 0.6
**Mn**	0.0–0.7	0.05–0.8	0.01–0.4	0.2 ± 0.2	0.3 ± 0.2	0.1 ± 0.1	0.004–0.620	0.001–0.186	0.02–0.07	0.1 ± 0.2	0.08 ± 0.06	0.05 ± 0.02
**Fe**^**2+**^	0.1–3.9	0.3–2.8	0.01–0.4	1.5 ± 1.3	1.6 ± 0.9	0.2 ± 0.1	0.2–3.7	0.3–2.0	0.2–1.0	0.7 ± 1.2	1.1 ± 0.7	0.6 ± 0.3
**Al**	0.2–16.3	0.7–19.3	0.2–1.6	5.2 ± 5.5	6.4 ± 5.7	0.7 ± 0.5	0.1–18.2	0.3–2.4	0.2–0.5	2.8 ± 6.3	1.2 ± 0.7	0.3 ± 0.1
**Pb [µg/l]**	0.0–5.5	0.05–9.2	0.0–3.6	2.5 ± 1.3	2.4 ± 2.5	2.1 ± 1.2	< 0.001–2.8	< 0.001–3.3	< 0.001–1.4	1.9 ± 0.9	1.6 ± 1.2	-

	**Tap water**		**Precipitation**		**Limit values**				
	***n* = 2**		***n* = 4**		**Namibian Water Act 54**				**WHO**
	**Ongwediva**	**Ruacana**	**Range**	**Mean ± sd**	**A**	**B**	**C**	**D**	
	**2017**	**2017**	**2018**	**2018**					
**Temp**	27.1	27.4	19.2–21.8	20.8 ± 1.0	-	-	-	-	-
**pH**	7.7	8.2	6.4–6.6	6.52 ± 0.05	6.0–9.0	5.5–9.5	4.0–11.0	4.0–11.0	-*
**EC**	105	97	17–404	152.3 ± 157.7	1500	3000	4000	> 4000	-*
**Turbidity**	1	0	1.4–22.2	12.7 ± 9.6	1	5	10	> 10	0.5
**Cl**^**−**^	5.2	3.7	26.9–72.1	49.5 ± 22.6	250	600	1200	> 1200	-*
**SO**_**4**_^**2−**^	< 1.0	< 1.0	< 1.0	-	200	600	1200	> 1200	-*
**NO**_**3**_^**−**^	< 0.01	< 0.01	1.3–35.2	13.8 ± 13.9	10	20	40	> 40	50
**NO**_**2**_^**−**^	< 0.01	< 0.01	0.07–0.62	0.3 ± 0.2	-*	-*	-*	-*	3
**NH4**	< 0.015	0.04	0.2–2.8	1.21 ± 1.06	1	2	4	> 4	-*
**PO**_**4**_^**3−**^	< 0.05	< 0.05	0.1–0.4	0.3 ± 0.1	-	-	-	-	-
**F**^**−**^	0.2	0.2	0.2–0.8	0.5 ± 0.3	1.5	2	3	> 3	1.5
**K**^**+**^	2.0	1.9	0.08–2.80	1.10 ± 1.08	0.2	0.4	0.8	> 0.8	-*
**Na**^**+**^	6.0	4.6	0.4- 63.7	20.8 ± 25.8	0.1	0.4	0.8	> 0.8	-*
**Mg**^**2+**^	2.2	1.8	0.2–2.7	1.2 ± 1.0	70	100	200	> 200	-*
**Ca**^**2+**^	7.0	8.7	1.0–17.0	6.3 ± 6.5	0.15	0.2	0.4	> 0.4	-*
**Mn**	0.0	0.0	0.004–0.071	0.03 ± 0.03	0.05	1	2	> 2.0	-*
**Fe**^**2+**^	0.0	0.0	0.02–0.27	0.14 ± 0.09	0.1	1	2	> 2.0	-*
**Al**	0.0	0.0	0.04–0.56	0.3 ± 0.2	0.15	0.5	1	> 1	0.9
**Pb [µg/l]**	2.0	9.5	< 0.001–1.0	-	50	100	200	200	10

^*^No guideline values have been established yet

#### The end of the dry season in 2017

Concerning physicochemical parameters temperature and pH, values are slightly higher in the Iishana than in the COC. All samples have alkaline pH values and are sorted into category A of the Namibian Water Act (6.00–9.00). Only site 17 is strongly alkaline with a value of 9 (category B). EC values of most of the Iishana belong to category A, while only sites 1, 5, 16, and 17 to category B. The COC always has values < 100 µS/cm and belongs to category A. The values for turbidity exceed the limit values at each site and are sorted into categories C and D. Only the tap water samples show turbidity values < 1 NTU. For turbidity, the WHO limit is 0.5 NTU and all sites exceed this guideline value.

The major anions of the Iishana and the COC can be ranked according to their dominance, based on means, in the order of the following: SO_4_^2−^  > Cl^−^  >  > NO_3_^−^  > F^−^ and the cations in the order of the following: Na^+^  > Ca^2+^  > Mg^2+^  > K^+^. Salts and salt compounds are dominant. The Ca^2+^ and Na^+^ values are completely in category D of the Namibian Water Act. Most of the element concentrations of tap water were below the detection limits, just Cl^−^ and F^−^ were detected in higher concentrations. In the Iishana and partially the COC, the striking parameters are Al and Fe^2+^, which occur in high amounts. The observed Fe^2+^ concentrations are considered a health threat for the population in 35.3% of the cases (14.3% for the COC, category D). Al is highly concentrated with a maximum of 16.3 mg/l at Iishana site 13 and a maximum of 18.2 mg/l at site 4 of the COC. Due to high aluminum levels, 70.6% of the Iishana sites and 5.9% of the canal-side sites are at increased health risk (category D). Within the study area, the spatial distribution of aluminum is eastbound, with higher values close to urban centers, like Oshakati and Ongwediva.

#### The end of the wet season in 2018

In 2018, sampling sites 2, 11, and 12 were dried up. The physicochemical parameters slightly changed compared to 2017. The pH value decreased by a value of 1 at the Iishana and 0.7 at the COC. Precipitation also has a slightly acid pH value with an average of 6.5. EC values decreased from an average of 911 to 615 µS/cm at the Iishana and 82 to 72 µS/cm at the COC. The precipitation shows values around 152 µS/cm, with a smaller range. Turbidity values increased on average from 631 to 722 NTU. In the COC, the change is even bigger with an average of 165 NTU (compared to 13 NTU in 2017). The temperature of the Iishana and the turbidity of the COC are significantly different between 2017 and 2018 (*p* = 0.04 and *p* = 0.005).

Like in 2017, the orders of anions in 2018 (SO_4_^2−^  > Cl^−^  >  > NO_3_^−^  > NO_2_^−^) and cations (Na^+^  > Ca^2+^  > Mg^2+^  > K^+^) for the Iishana and the COC (Ca^2+^  > Na^+^  > Mg^2+^  > K^+^) show a dominance of salts and salt compounds. Anions in precipitation can be ordered: Cl^−^  >  > NO_3_^−^  >  > F^−^  > NO_2_^−^ and cations: Na^+^  > Ca^2+^  > Mg^2+^  > K^+^. From 2017 to 2018, the concentrations of SO_4_^2−^, Cl^−^, and NO_3_^−^ increased at sites 13, 14, and 16 from category A to B or C and decreased at others (3, 5) from B to A. For the COC, no changes were observed for these parameters. Ca^2+^ and Na^+^ are still in category D and most of the sites have increased values. Mn and NO_2_^**−**^ increased at sites 3, 13, and 14. PO_4_^3−^ and NH_4_^+^ increased at sites 13, 14, and 16 from not dangerous concentrations (categories A and B) to concentrations considered as high health risk (category D). The Mn concentrations of the Iishana have been a potential threat to human health in both years (category B and C). Only some sites at the COC included Mn in these amounts (sites 4, 6, 20, 24, and 25). Significant differences were found between the Iishana for Ca^2+^ (*p* = 0.04), NH_4_^+^ (*p* = 0.02), NO_3_^**−**^ (*p* = 0.01), Al (*p* = 0.005), and Fe^2+^ (*p* = 0.03). For the COC: Cl^**−**^ (*p* = 0.02), Pb (*p* = 0.01), NO_3_^**−**^ (*p* = 0.0009), F^**−**^ (*p* = 0.002), and Al (*p* = 0.02). Between the end of the dry season in 2017 and the end of the rainy season in 2018, mixing of the Iishana water with rainwater and thus a dilution could be expected. Some parameters showed decreasing concentrations: pH, EC, Na^+^, Cl^−^, F^−^; however, turbidity as well as K^+^, Mg^2+^, Ca^2+^, SO_4_^2−^, Fe^2+^, NO_3_^−^, NO_2_^−^, and Al increased.

Concentrations of NO_3_^−^, NO_2_^−^, PO_4_^3−^, and SO_4_^2−^ show great spatial variability. Sites that are close to settlements or cities, in the eastern part of the study area, around the city of Oshakati, or along the COC (3, 17, 27, and 28), show higher values than remote sites in the southwestern part (11, 12, and 22). Sites north of road dams (retention of flood waters and preferred sediment accumulation) also show higher concentrations of metals and nutrients (16, 18).

In 2018, the highest aluminum concentration of 19.3 mg/l and an average of 6.4 mg/l were analyzed, an increase of 23% compared to 2017. In total, 78.6% of the Iishana and 55.5% of the COC represent a high risk for human health. Especially the Iishana close to Oshakati (sites 3 and 5) show high aluminum values. Site 4 at the COC contained more than 18 mg/l in 2017 but decreased in 2018. Concerning Fe^2+^, 35.7% of the Iishana and 22.2% of the COC samples are unsuitable for human consumption and represent a high health risk (category D). Light metals are present in precipitation samples with low concentrations close to the detection limit.

#### The end of the rainy season in 2019

Due to low rainfall during the rainy seasons of 2018 and 2019 and ongoing high evapotranspiration values, several Iishana completely lost their water. The pH values of the Iishana increased on average to 8.1; in the COC, they dropped to the neutral value of 7. The EC values of the Iishana increased on average to 725 µS/cm and had a broader range from 76 to 2935 µS/cm. The COC also had a wide range from 64 to 109 µS/cm and raised on average to 75 µS/cm. Turbidity exceeds in every sample the limit of 1 NTU. Over the entire observation period, EC values of the Iishana belong to categories A and B and represent water, which still requires prior treatment before consumption.

The order of anions in the Iishana is, like in 2017, SO_4_^2−^  > Cl^−^  >  > NO_3_^−^  > F^−^ and of cations: Na^+^  > Ca^2+^  > Mg^2+^  > K^+^. For the COC, the cations composition changed: Ca^2+^  > Na^+^  > Mg^2+^  > K^+^. The high Al and Fe^2+^ values in the Iishana water of the last 2 years were not reached in this campaign. In 2019, 0.7 mg/l was detected on average. The maximum was found at site 7 with 1.6 mg/l. In the center of the study area (northwest to southeast), there are several sites with high Al concentrations (sites 18, 9, 13, 14, and 11). The Fe^2+^ concentrations have decreased and samples of the Iishana and the COC are in categories A, B, or C. Heavy metals were found in trace concentrations at all sites and fell every year in category A. Significant differences to 2017 and 2018 could be tested for the Iishana and the COC for several parameters, like Al (*p* = 0.018), Ca^2+^ (*p* = 0.042), and Fe^2+^ (*p* = 0.03) (more information can be found in the supplementary data 3).

#### Comparison of the three campaigns

**Fig. 3:**
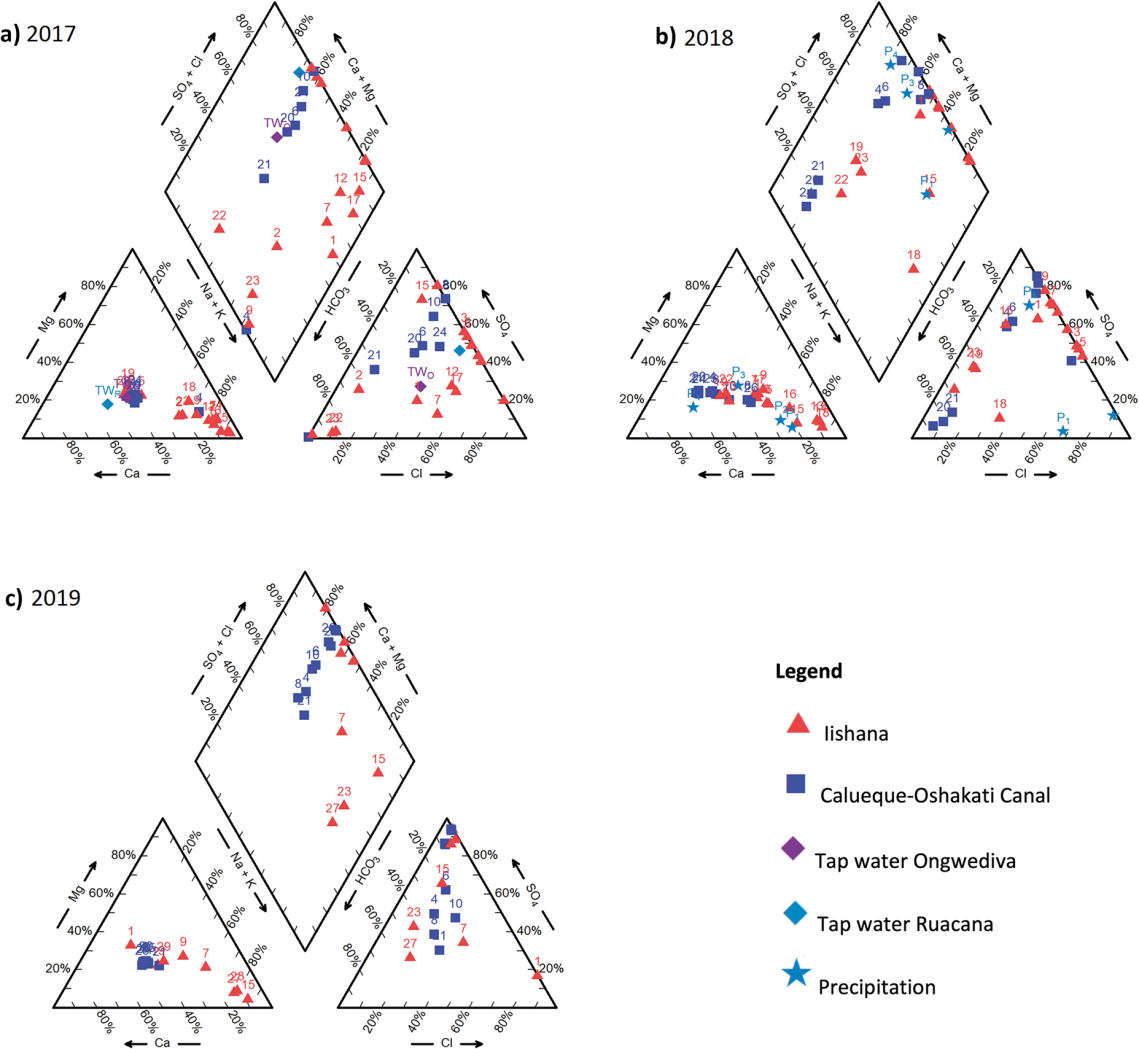
Piper diagrams of the Iishana, the Calueque-Oshakati canal, tap water, and precipitation samples from 2017 to 2019

To illustrate the ionic composition of the water samples over all 3 years, Piper diagrams were created (see Fig. 3a–c). For this presentation, hydrogen carbonate (HCO_3_) values were calculated via the ion balance. In 2017 (Fig. 3a), most of the Iishana are Na–K dominated. The COC, the tap water, and Iishana 5, 19, and 22 contain more calcium. The sites scatter much more in the anion content. Sulfate and chloride contents differ extremely between the sites. In 2018 (Fig. 3b), the calcium dominance increased, the chloride content decreased, while sulfate and bicarbonate dominated. Alkaline earths dominate 2018 more than in 2017, and sulfate and chloride prevail slightly more than hydrogen carbonate.

In 2019 (Fig. 3c), the ratio of the cations has hardly changed and the anions are more dominated by sulfate and hydrogen carbonate. Earth alkaline, sulfate, and chloride prevail, although the sites of the COC are even more homogeneous.

Statistically significant effects between the three campaigns exist for the Iishana and the COC for almost all parameters, except EC, Mn, and SO_4_^2−^ (a detailed table can be found in the supplementary data 4).

**Fig. 4:**
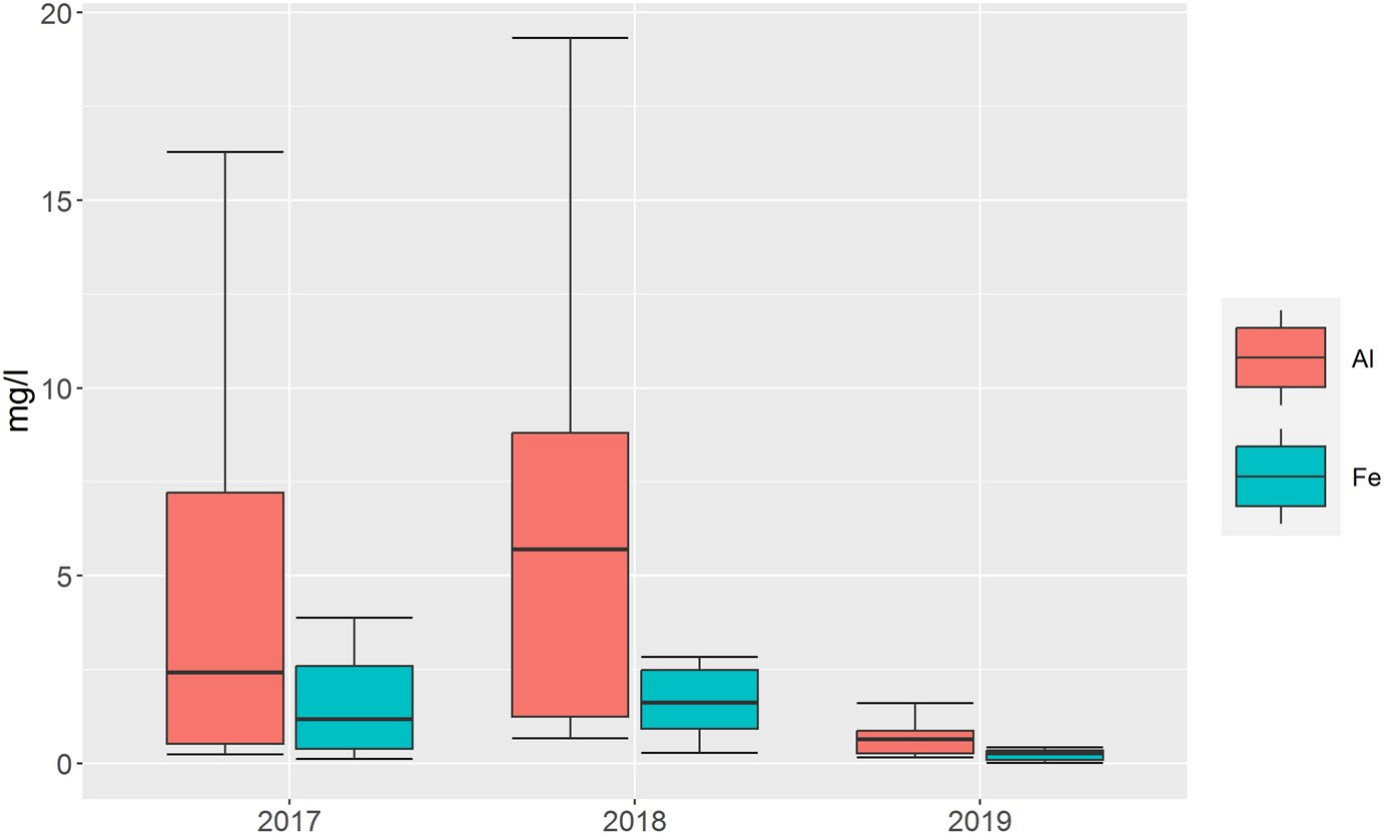
Changes in Al and Fe concentrations in the Iishana between 2017 and 2019

The aluminum concentrations of the 3 years are an example of the spatial distribution of the water quality in the study area. High values in the eastern part, around the urban agglomeration of the city Oshakati, are striking. Changes over the 3 years are particularly visible at the Iishana (*p* = 0.005). Except for sites that dried up over the 3 years, most values increased from 2017 to 2018 and decreased again in 2019. Along the COC, the values increase with the flow direction. Maximum values were measured in 2018 and at site 4 in 2017, and differences were significant (*p* = 0.02). Figure 4 shows an example of the changes in Al and Fe^2+^ for the Iishana over the study period.

### Suspended solids and sediments

Suspended solids and sediments are presented in Table 2. Ranges, means, and standard deviations of physicochemical parameters and main concentrations of the Iishana and the COC show slight changes over the 3 years. PH values and EC could only be measured for the bottom sediments of the dried Iishana or the canal, not for the suspended solids in the water. The pH values of the Iishana are slightly alkaline on average, with a strong alkaline maximum of 9.3. The COC has lower pH values with 7.5. As with the water samples, the sediments of the Iishana show up to 14,010 µS/cm, much higher EC values than the canal with 104 µS/cm.

**Table 2:** Range, mean, and standard deviation (sd) for physio-chemical parameters and major element concentrations of suspended solids and sediments of the Iishana, the COC, and the waterworks in Oshakati (WO). EC in microsiemens per centimeter, concentrations in milligrams per gram, and Pb in micrograms per gram

	**Iishana**									
	**Suspended solids < 0.45 µm**						**Sediments < 0.063 mm**		**Sediments < 1.00 mm**	
	***n* = 8, 9, 5**						***n* = 17**		***n* = 17**	
	**Range**			**Mean ± sd**			**Range**	**Mean ± sd**	**Range**	**Mean ± sd**
	**2017**	**2018**	**2019**	**2017**	**2018**	**2019**	**2019**	**2019**	**2019**	**2019**
**pH**	-	-	-	-	-	-	6.5–9.3	7.4 ± 0.8	-	-
**EC**	-	-	-	-	-	-	80–14,010	2648 ± 3941	-	-
**K**^**+**^	5.5–18.0	3.9–18.8	4.1–10.8	12.8 ± 4.3	11.5 ± 5.1	7.0 ± 2.2	4.6–15.9	10.1 ± 3.5	0.3–16.2	5.5 ± 4.4
**Na**^**+**^	0.9–5.1	4.4–12.5	2.4–10.9	2.2 ± 1.1	8.3 ± 2.5	5.5 ± 3.1	0.6–62.7	10.4 ± 14.4	0.2–43.4	6.3 ± 11.4
**Mg**^**2+**^	8.1–10.6	3.3–11.4	7.7–9.1	9.4 ± 0.8	8.3 ± 2.4	8.4 ± 0.5	6.2–14.1	9.6 ± 1.9	0.4–10.3	5.3 ± 3.8
**Ca**^**2+**^	0.5–3.0	1.2–3.3	1.8–9.7	1.3 ± 0.7	2.6 ± 0.7	5.8 ± 2.5	1.6–14.7	6.3 ± 3.7	0.7–7.1	3.1 ± 1.9
**Mn**	0.2–0.3	0.07–0.31	0.2–0.3	0.22 ± 0.03	0.2 ± 0.1	0.22 ± 0.04	0.3–0.6	0.38 ± 0.08	0.02–0.40	0.2 ± 0.1
**Fe**^**2+**^	27.8–44.6	11.0–41.2	19.6–30.2	39.0 ± 4.9	30.5 ± 9.3	26.4 ± 4.0	12.4–38.0	30.8 ± 7.0	1.0–37.8	16.8 ± 12.1
**Al**	58.9–86.7	25.8–85.2	56.6–70.4	80.7 ± 8.9	64.0 ± 18.3	64.6 ± 5.2	27.0–77.6	63.5 ± 13.9	1.2–75.4	35.2 ± 26.2
**Pb [µg/g]**	6.9–12.6	4.0–9.1	12.5–228.2	9.3 ± 2.0	7.0 ± 1.3	82.7 ± 81.6	0.009–0.018	0.013 ± 0.003	0.001–0.012	0.007 ± 0.004

	**Calueque-Oshakati canal**					**Waterworks Oshakati**
	**Suspended solids < 0.45 µm**	**Sediments < 0.063 mm**		**Sediments < 1.00 mm**		**Sediment < 0.063 mm**
	***n* = 1**	***n* = 2**		***n* = 2**		***n* = 1**
		**Range**	**Mean ± sd**	**Range**	**Mean ± sd**	
	**2017**	**2019**	**2019**	**2019**	**2019**	**2019**
**pH**	-	6.8–7.5	7.1 ± 0.4	-	-	6.1
**EC**	-	50–104	77 ± 27	-	-	423.0
**K**^**+**^	11.3	4.1–4.4	4.2 ± 0.1	0.1–0.2	0.19 ± 0.05	2.4
**Na**^**+**^	1.7	0.6–1.3	0.9 ± 0.4	0.11–0.12	0.118 ± 0.004	0.5
**Mg**^**2+**^	9.2	6.8–7.2	7.0 ± 0.2	0.3–0.4	0.33 ± 0.04	3.4
**Ca**^**2+**^	1.3	7.9–13.7	10.8 ± 2.9	1.3–1.8	1.5 ± 0.2	3.7
**Mn**	0.2	0.6–1.1	0.8 ± 0.2	0.032–0.039	0.035 ± 0.004	0.2
**Fe**^**2+**^	38.4	25.8–28.6	27.2 ± 1.4	1.0–1.5	1.3 ± 0.2	31.6
**Al**	75.6	56.2–57.7	56.9 ± 0.8	1.0–2.3	1.7 ± 0.7	79.9
**Pb [µg/g]**	14.7	0.01–0.02	0.017 ± 0.003	0.001–0.002	0.002 ± 0.000	0.01

The cations for the suspended solids of the Iishana water can be ordered: Mg^2+^  > K^+^  > Ca^2+^  > Na^+^. For the COC: Mg^2+^  > K^+^  > Na^+^  > Ca^2+^. Concentrations of K^+^, Al, and Fe^2+^ decreased from 2017 to 2019, while Na^+^ and Mg^2+^ decreased from 2017 to 2018 and increased from 2018 to 2019. Only Ca^2+^ increased from 2017 to 2019. Concentrations of Al vary between 25.8 to 86.7 mg/g and of Fe^2+^ between 11.0 and 44.6 mg/g. In the COC, the value of Al went up to 75.6 mg/g and Fe^2+^ to 38.4 mg/g. Significant differences between 2017 and 2018 were confirmed for Na^+^ (*p* = 0.007), Ca^2+^ (*p* = 0.003), and Fe^2+^ (*p* = 0.04).

The ion concentrations of the sediments are similar to those of suspended solids. In the fraction < 0.063 mm, the cations for the Iishana can be ordered: Na^+^  > K^+^  > Mg^2+^  > Ca^2+^. For the COC, Ca^2+^  > Mg^2+^  > K^+^  > Na^+^. The fraction < 1.00 mm shows the same distribution but in lower concentrations. The light metals Al and Fe^2+^ accumulated stronger in the smaller fraction < 0.063 mm than in the fraction < 1 mm (Al: 63.5 mg/g in the Iishana and 56.9 mg/g in the COC, Fe^2+^: 30.8 mg/g in the Iishana and 27.2 mg/g in the COC on average).

Almost no organic material could be found in the Iishana sediment samples and the water content was low, except for the sample of the sludge from the COC water treatment at the waterworks in Oshakati. This sample contains nearly the same Al and Fe^2+^ concentrations as the sediments of the Iishana and the COC: Al with 79.9 and Fe^2+^ with 31.6 mg/g. Further heavy metal concentrations can be found in the supplementary data 3.

## Discussion

### Calueque-Oshakati canal

The Calueque-Oshakati canal is fed by the Calueque reservoir in Angola, which receives water from the Kunene River. Several parameters (EC, Ca^2+^, K^+^, Mg^2+^, and Na^+^) are different from the Iishana, but comparable to other studies in the region (Koeniger et al., 2020; Shuuya & Hoko, 2014). Both studies and the present study discovered an increase in pollution in the course of the COC and found the highest concentrations near the urban area of Oshakati, which is located at the end of the canal. Especially, site 4 is striking with very high Al and Fe^2+^ concentrations in the water column, the suspended solids, and the sediments. Additional samples upstream and downstream of site 4 contain high Al concentrations as well. Even the Olushandja reservoir contains up to 0.3 mg/l Al, and because of the absence of point sources, a diffuse input has to be assumed. Local waterworks use aluminum hydroxide chloride (aluminum chlorohydrate coagulant Ultrafloc 3200) as a hydrolyzing flocculant in the flocculation process (Shuuya & Hoko, 2014). Hydrolysis of the dissolved salts of the trivalent aluminum ions produces metal hydroxides, which are necessary to destabilize the dispersed substances (Cañizares et al., 2008; Lin et al., 2008). After flocculation, the aluminum residues remain in the sludge, which is dried in the waterworks and then passed to local farmers as potential fertilizer. The sample of the sludge is contaminated with Al and Fe^2+^. As there is no local industry that uses aluminum in high amounts (NAMF, 2017), the source could be either illegal waste disposal or pollution sources on fields, like the sludge, from which aluminum might be washed into the canal by surface runoff during rain events. The canal is exposed to various pollutant sources and water-extracting devices, like animals, the use of pumps, washing, and bathing. In addition, farmers take their irrigation water from the canal and the Olushandja dam (Fiebiger et al., 2010). The extensive use of water, in particular during the dry season, causes large water level fluctuations in the reservoir. During floods, the open canal is not protected against flooding water and many pollutants from surrounding settlements are washed in. Its use without treatment is limited due to its hydrochemical properties.

### The Iishana system

The surface water from the Iishana is limited in its quality. Constituents that are striking are EC, turbidity, Al, Fe^2+^, Ca^2+^, and Na^+^. Wanke et al. (2014) showed comparable results for EC and turbidity in a study of the quality of hand-dug wells (shallow perched aquifers). They found similar high EC and turbidity values at the end of the rainy season in 2010. In the neighboring Okavango Delta, EC is smaller than 200 µS/cm (Mmualefe & Torto, 2011) and lower than in the CB. Mmualefe and Torto (2011) measured low EC values in the Okavango Delta in 2010, which they explain by the leakage of salts into underground aquifers. High evaporation results in water loss and a concentration of dissolved salts in the remaining water and sediments (Zimmermann et al., 2006). Since near-surface groundwater is very saline in the Iishana system, accumulated salts at the surface may leak into underground aquifers (McCarthy & Metcalfe, 1990). Endorheic systems are often saline, because of concentration processes due to evaporation (Yapiyev et al., 2017). In hand-dug wells and boreholes, which are groundwater fed, the elements F^−^, NO_3_^−^, PO_4_^3−^, and SO_4_^2−^ were detected in higher concentrations than in Iishana in the present study (Wanke et al., 2014, 2017). A leakage from Iishana into aquifers could cause higher concentrations in underlying groundwater horizons than in surface waters. Shanyengana et al. (2004) showed that groundwater and surface water are influenced by seasonal trends. Several processes, like concentration due to evaporation, dissolution of saline sediments (mainly evaporites), mixing with older and more saline groundwater, and precipitation influence the major-ion composition. Rainfall events can refresh surface waters and increase water quality. The rainfall during the study period was low but could cause a dilution and lower concentrations. The precipitation gradient over the study area is depicted in the spatial variations of the results. In the eastern part, with higher rainfalls, less Iishana had dried up between 2017 and 2018. Until 2019, even more Iishana dried up and the water levels decreased rapidly. Between the dry season in 2017 and the rainy/wet seasons in 2018 and 2019, significant differences for several parameters were proved. The missing dilution by too less rainfall could cause the increased salt contents (Na^+^ and Ca^2+^).

All samples show elevated TC and TOC concentrations, which indicate a high level of organic compounds (supplementary data 3). The corresponding high TNb concentrations cause an increased primary production. The high redox potential of the samples acts as an oxidizing agent and favors oxidized compounds, like nitrate, sulfate, Fe, and Mn oxides. Warm water temperatures, around 25 °C in September and 27 °C in March/April favor the spreading of bacteria. The availability of oxygen is largely responsible for the presence of bacteria; low concentrations cause high bacterial counts due to consumed oxygen. Microorganisms adhere to and multiply at the dissolved solids, which offer a suitable environment for organisms (Liu et al., 2016; Luo et al., 2019). The differing oxygen saturation values at several sites suggest the different abundances of consumers. The blue-green algae produce several types of toxins, which can cause health risks for humans. Algae blooms intensify with an increasing eutrophication rate (O’Neil et al., 2012). This increasing eutrophication rate is indicated by temperature, salinity, chlorophyll-α, dissolved oxygen, nutrients, and water transparency (Deggobis et al., 2000). The presented bacteriological risk for human consumption was also identified in hand-dug wells by McBenedict et al. (2017). In their study of hand-dug wells in the Iishana region, they found several bacteria of the *Bacillus* genus. Some of the species are pathogenic and can cause gastrointestinal diseases (McBenedict et al., 2017).

Close to the urban area of Oshakati and Ongwediva anthropogenic influences are prevalent: Wastewater is discharged to the surface waters, more litter is distributed, and the waste disposal site, without a filtration system, is located close to water sources. Sites 9, 16, and 18 show high concentrations of metals and nutrients although not located near settlements. However, these sites are situated upstream of channel crossing road dams, which could have a water retention effect during flood events (Arendt et al., 2020) resulting in the accumulation and concentration of sediments and pollutants. Some Iishana (sites 3, 5, 19) are located close to the canal and show similarly very low concentrations. Residents reported that they pipe water from the canal into the Iishana to provide water for their animals. This mixing could result in dilution effects in the Iishana.

Further metals, like As, Cd, Co, Cr, Cu, Ni, Sr, and Zn, are detectable in low concentrations (see supplementary data 3). However, it can be assumed that some sediments from the planalto in Angola were transported by surface runoff and accumulated in the southern part of the Iishana system, as it last happened during the flood in 2011 (Persendt et al., 2015).

High concentrations of Al in the water column, the suspended solids, and the sediments indicate a long-lasting source of anthropogenic influence (Power & Chapman, 1992). Since the free water column also has high concentrations of aluminum, sedimentation processes must have taken place over long periods. As there is only a small metal-processing industry in the region (NAMF, 2017) and the geogenic background (concentrations in soil or water that are due to natural processes) does not show elevated aluminum concentrations (Bäumle & Himmelsbach, 2018; Dill et al., 2013), there must be other local sources. The high Al concentration in the sample of the sludge of 79.9 mg/g could be a reason for the increased Al content in the Iishana and some sections of the canal. It is possible that aluminum from the sludge on the field was washed out by precipitation, diluted, and accumulated in sediments and water of the Iishana. However, there is no information available on when the use of the sludge as potential fertilizers was initialized and whether all four waterworks along the canal hand over the sludge to farmers.

Iron concentrations are also outstandingly high, in all three measurement campaigns. Li et al. (2018b) reported generally large concentrations of Fe^2+^ in the groundwater in northern Namibia, although the geogenic background in Namibia does not contain much iron (Bäumle & Himmelsbach, 2018; Dill et al., 2013). Locally, there is no iron processing industry (NAMF, 2017); therefore, the iron concentrations measured in this study must come from other local point sources. Similar to aluminum hydroxide chloride or aluminum sulfate, iron chloride or iron sulfate is used for flocculation in waterworks (Aboulhassan et al., 2006).

Sediments and suspended solids show similar values, in particular for Al and Fe^2+^. It is known that loads in bottom sediments are often higher than in suspended solids or the water column (Power & Chapman, 1992). The results from the water column, suspended solids, and sediments indicate that the pollutants have accumulated in the sediments over a long period.

All the samples, except for the tap water, have concentrations that exceed the limits of the Water Act 54 and the WHO guidelines, especially turbidity, Al, Fe^2+^, Ca^2+^, and Na^+^. The metals Al and Fe^2+^ do not directly affect human health but can cause intoxication over a long period. Turbidity directly threatens the health of the local population. High turbidity values are an indicator for suspended and dissolved solids, which favors the accumulation of pollutants. If it is unclear, which specific substances cause the high turbidity, it is not recommended to consume this water.

Differences between Iishana and the COC are significant for several parameters (see supplementary data 4) since water is from different sources. Water from the COC comes from the Kunene River in Angola, i.e., from another hydrological system. As part of a running water system, it has a continuous freshwater supply. Due to the open canal, anthropogenic pollution is present. The water from the Iishana is originally rainwater and is only connected to a hydrological system with freshwater supply in case of extreme rainfall events or during floods. This explains the huge differences in terms of water quality and usability. At the end of the dry season in 2017, the surface water has been without any exchange for six months. It has been affected by intensive use and did not have any chance to renew. During the wet seasons of 2018 and 2019, the little amount of rainfall could not cause dilution. These seasons have rather led to a continuation of the drought period that started in 2015 and was only finished with substantial rainfall during the rainy season of 2019/2020.

This study provided much important information about the water quality of the Iishana system. As one of the main water sources for the local population in the region, it is essential to gain more knowledge about the ecosystem of the Iishana; however, the study has some limitations. The little amount of rainfall in 2018 and 2019 that results in a continuation of the drought period since 2015 impedes the assessment of the water quality during that period. Nevertheless, the results indicate the quality of the Iishana water and sediments decreases with the increase of electrical conductivity, turbidity, and Al and Fe^2+^ concentrations during a drought period. Missing mixing of water, high temperatures, and evaporation create optimal conditions for bacteria. The accumulation of pollutants and harmful substances is a consequence. Higher temporal and seasonal resolutions are required to consolidate these results. Dried up Iishana in 2019 result in low sample numbers, which in turn impedes statistical testing. Therefore, certain statistically significant differences may not have been detected.

## Conclusion and outlook

Drought periods have a lasting effect on the water quality of surface waters, increased salt accumulations, and turbidity values. The results show that the water of the Iishana is subject to these processes and is exposed to more pollution sources with years of low precipitation. Critical concentrations could be detected for turbidity, Na^+^, Ca^2+^, Al, and Fe^2+^. Parameters, like Cl^−^, SO_4_^2−^, Mn, and NO_3_^−^, showed elevated concentrations. Several heavy metals are present in harmless concentrations. The physicochemical conditions, combined with the relatively high carbon and nitrogen levels, indicate organic compounds and favor primary production. Local characteristics of the individual locations, such as proximity to settlements, strengthen spatial differentiation. Changes over the 3 years of investigation are evident, partially significant, and can be attributed to different states of water availability, which underlines that prevailing weather conditions are important for the quality of water resources.

Important results on the water quality in the Iishana system in the CB are provided and contribute to a better understanding of the ecosystem. The results of the analyses of hydrochemical parameters and ion concentrations of the water show that consumption is not recommended without prior treatment. Further studies on the composition of the waterworks’ flocculation sludge and the potential input of Al and Fe^2+^ into the fields are necessary to identify and reduce the sources of the metals. An implementation of desalination and water treatment methods would be necessary (Lux & Janowicz, 2009; McBenedict et al., 2017).

Future climatic projections show a decrease in rainfall and an increasing frequency of drought periods (Kundzewicz et al., 2014; Luetkemeier & Liehr, 2015; Masih et al., 2014; Ujeneza & Abiodun, 2015). In combination with an increasing potential evaporation, these conditions likely result in reduced water availability (Angula & Kaundjua, 2016; Archer et al., 2018; Engelbrecht et al., 2009). It is important to find solutions for effective water storage, particularly during the dry seasons (Arendt et al., 2021). People are dependent on the water of the Iishana, and this dependence will increase in the coming years as the demand for water increases due to projected population growth. Thus, the presented water-related challenges, e.g., water quality and water availability, will further increase in the future and the development of adequate water treatment techniques as well as water resource protection measures need to be understood as a priority task.

## Supplementary Information

Below is the link to the electronic supplementary material.

Supplementary file1 (DOCX 35 KB)

## Data Availability

The data set collected within the scope of this study will be published and accessible at GFZ Data Services. Additional data are properly cited and referred to in the reference list.
