# Supplementary material for: Water and sediment pollution of intensively used surface waters during a drought period — a case study in Central Northern Namibia
Source: Environ Monit Assess. 2023 Jul 6;195(8):924. doi: 10.1007/s10661-023-11505-1 (PMC10326107; doi:10.1007/s10661-023-11505-1)
Supplement: Supplementary file 1 — Supplementary file1 (DOCX 35 KB) [file 10661_2023_11505_MOESM1_ESM.docx]

**Water and sediment pollution of intensively used surface waters during a drought period – a case study in Central Northern Namibia**

*Environmental Monitoring and Assessment*

Leona Faulstich, Robert Arendt, Christian Reinhardt-Imjela, Achim Schulte, Joachim Lengricht, Petrina Johannes

Freie Universität Berlin, leona.faulstich@fu-berlin.de

**Supplementary data 1**

General information about applied methods.

| **sample** | **sample preparation** | **method** | **parameters** | **accuracy** | **test protocol** |
| --- | --- | --- | --- | --- | --- |
| water | *in situ* measurement | YSI-multiparameter probe  6600 V2-4 | temperature,  pH-value,  redox potential,  oxygen content,  oxygen saturation,  electrical conductivity,  turbidity,  chlorophyll-α,  cyanobacteria | ± 0.15 °C  ± 0.2 units  ± 20 mV in standard  ± 2 % of value  ± 2 % of value  ± 0.5 % of value  ± 0.3 NTU  linearity R^2^ = 0.9999  linearity R^2^ = 0.9999 | - |
| water | centrifugation  (20 min at 6000 min^-1^)  filtration  (syringe filter holders, 0.45 µm) | portable HACH DR 1900 VIS spectrophotometer | Cl^-^, F^-^, NH_4_^+^, NO_3_^-^, NO_2_^-^, PO_4_^3-^, SO_4_^2-^, COD, TNb, TC, TIC, TOC | 340 - 800 nm | - |
| water | filtration (syringe filter holders, 0.45 µm), acidification (0.1 ml of nitric acid), cooled transport in PE bottles;  centrifugation  (15 min at 10,000 min^-1^)  filtration  (membrane cellulose acetate filter, 0.45 µm) | ICP-OES 2000 | Al, As, Cd, Ca^2+^, Cr, Co, Cu, Fe^2+^, Pb, Mg^2+^, Mn, Ni, K^+^, Na^+^, Sr, Zn | 1 - 3 % for main elements,  5 - 20 % relative for trace elements | DIN EN ISO 11885:2009 |
| water – suspended solids | cooled transport in PE bottles;  centrifugation  (15 min at 10,000 min^-1^)  filtration  (membrane cellulose acetate filter, 0.45 µm)  sample of > 0.5 g | digestion with aqua regia  ICP-OES 2000 | Al, As, Cd, Ca^2+^, Cr, Co, Cu, Fe^2+^, Pb, Mg^2+^, Mn, Ni, K^+^, Na^+^, Sr, Zn | 1 - 3 % for main elements,  5 - 20 % relative for trace elements | DIN EN 16174:2012-11  DIN EN ISO 11885:2009 |
| sediment | transport;  homogenization,  drying for 24 h at 45 °C,  sieving:  2 mm, 1 mm, 0.063 mm |  |  |  | DIN EN ISO 66165-2:2016-08 |
| sediment | transport;  homogenization,  drying for 24 h at 45 °C,  sieving:  2 mm, 1 mm, 0.063 mm | grain size distribution  Beckman Coulter LS 13 320 laser diffractometer | grain sizes:  clay, silt, sand |  | ISO 13320:2020-01  DIN EN ISO 14688-1:2018-05 |
| sediment fraction  1-2 mm | sieving | pH-value and EC measurement | pH  EC |  | DIN EN 15933:2012-11  DIN EN 27888:1993-11 |
| sediment fraction  1-2 mm | sieving | determination of TC  LECO TruSpec Elemental Determinator | TC |  | DIN EN 15936:2012-11 |
| sediment fraction  1-2 mm | sieving | determination of TIC  Carmhograph C16 | TIC |  | DIN EN 15936:2012-11 |
| sediment fraction 0.063-1 mm | wet sieving | digestion with aqua regia  ICP-OES 2000 | Al, As, Cd, Ca^2+^, Cr, Co, Cu, Fe^2+^, Pb, Mg^2+^, Mn, Ni, K^+^, Na^+^, Sr, Zn | 1 - 3 % for main elements,  5 - 20 % relative for trace elements | DIN EN 16174:2012-11  DIN EN ISO 11885:2009 |
| sediment fraction < 0.063 mm | wet sieving | digestion with aqua regia  ICP-OES 2000 | Al, As, Cd, Ca^2+^, Cr, Co, Cu, Fe^2+^, Pb, Mg^2+^, Mn, Ni, K^+^, Na^+^, Sr, Zn | 1 - 3 % for main elements,  5 - 20 % relative for trace elements | DIN EN 16174:2012-11  DIN EN ISO 11885:2009 |

**Water and sediment pollution of intensively used surface waters during a drought period – a case study in Central Northern Namibia**

*Environmental Monitoring and Assessment*

Leona Faulstich, Robert Arendt, Christian Reinhardt-Imjela, Achim Schulte, Joachim Lengricht, Petrina Johannes

Freie Universität Berlin, leona.faulstich@fu-berlin.de

**Supplementary data 2**

General information of the samples: table differentiated by sampling site.

| **water** |  |  |  |  |  |  |  |  |  |  |
| --- | --- | --- | --- | --- | --- | --- | --- | --- | --- | --- |
| **name** | **system** | **region** | **location** | **classification** | **water level** | | | **water depth [m]** | | |
|  |  |  |  |  | **2017** | **2018** | **2019** | **2017** | **2018** | **2019** |
| **1** | Oshana | Oshana region | 0.15 m surface water | rural | water | water | water | 0.4 | > 0.7 | 0.6 |
| **2** | Oshana | Oshana region | 0.15 m surface water | rural | water | dry | dry | > 0.7 | // | // |
| **3** | Oshana | Oshana region | 0.15 m surface water | rural | water | water | dry | 1.0 | 1.4 | // |
| **4** | Calueque-Oshakati canal | Oshana region | 0.15 m surface water | rural | water | water | water | 0.5 | > 0.7 | > 0.7 |
| **5** | Oshana | Omusati region | 0.15 m surface water | rural | water | water | dry | > 0.7 | > 0.7 | // |
| **6** | Calueque-Oshakati canal | Omusati region | 0.15 m surface water | rural | water | water | water | > 0.7 | > 0.7 | > 0.7 |
| **7** | Oshana | Omusati region | 0.15 m surface water | rural | water | water | water | > 0.7 | > 0.7 | 0.3 |
| **8** | Calueque-Oshakati canal | Omusati region | 0.15 m surface water | rural | water | water | water | > 0.7 | > 0.7 | > 0.7 |
| **9** | Oshana | Omusati region | 0.15 m surface water | rural | water | water | water | > 0.7 | > 0.7 | 0.6 |
| **10** | Calueque-Oshakati canal | Omusati region | 0.15 m surface water | rural | water | water | water | 1.2 | > 0.7 | > 0.7 |
| **11** | Oshana | Oshana region | 0.15 m surface water | rural | water | dry | dry | > 0.7 | // | // |
| **12** | Oshana | Omusati region | 0.15 m surface water | rural | water | dry | dry | > 0.7 | // | // |
| **13** | Oshana | Omusati region | 0.15 m surface water | rural | water | water | dry | > 0.7 | 0.3 | // |
| **14** | Oshana | Omusati region | 0.15 m surface water | rural | water | water | dry | > 0.7 | > 0.7 | // |
| **15** | Oshana | Ohangwena region | 0.15 m surface water | rural | water | water | water | > 0.7 | > 1.9 | > 0.8 |
| **16** | Oshana | Omusati region | 0.15 m surface water | rural | water | water | dry | > 0.7 | > 1.4 | // |
| **17** | Oshana | Omusati region | 0.15 m surface water | rural | water | water | dry | 0.5 | 1 | // |
| **18** | Oshana | Omusati region | 0.15 m surface water | rural | water | water | dry | > 0.7 | > 0.7 | // |
| **19** | Oshana | Omusati region | 0.15 m surface water | rural | water | water | water | > 0.7 | > 0.7 | > 0.7 |
| **20** | Calueque-Oshakati canal | Omusati region | 0.15 m surface water | rural | water | water | water | > 0.7 | > 0.7 | > 0.7 |
| **21** | Calueque-Oshakati canal | Omusati region | 0.15 m surface water | rural | water | water | water | > 2.5 | 0.5 | > 0.7 |
| **22** | Oshana | Omusati region | 0.15 m surface water | rural | water | water | dry | > 0.7 | 0.2 | // |
| **23** | Oshana | Omusati region | 0.15 m surface water | rural | water | water | water | > 0.7 | > 0.7 | 0.7 |
| **24** | Calueque-Oshakati canal | Omusati region | 0.15 m surface water | rural | water | water | water | > 1.5 | > 0.7 | > 0.7 |
| **25** | Calueque-Oshakati canal | Oshana region | 0.15 m surface water | rural | // | water | water | // | > 0.7 | > 0.7 |
| **26** | Calueque-Oshakati canal | Oshana region | 0.15 m surface water | rural | // | water | water | // | > 0.7 | > 0.7 |
| **27** | Oshana | Oshana region | 0.15 m surface water | rural | // | // | water | // | // | > 0.7 |
| **29** | Oshana | Oshana region | 0.15 m surface water | rural | // | // | water | // | // | > 0.9 |
| **TW Ongwediva** | water supply | Oshana region | // | urban | water | // | // | // | // | // |
| **TW Ruacana** | water supply | Omusati region | // | urban | water | // | // | // | // | // |
| **Precipitation** | // | Oshana region | // | urban | // | water | // | // | // | // |
| **Precipitation** | // | Oshana region | // | urban | // | water | // | // | // | // |
| **Precipitation** | // | Oshana region | // | urban | // | water | // | // | // | // |
| **Precipitation** | // | Oshana region | // | urban | // | water | // | // | // | // |

// = no measurement possible

| **sediments** |  |  |  |  |  |
| --- | --- | --- | --- | --- | --- |
| **name** | **system** | **region** | **location** | **classification** | **water level** |
| **3** | Oshana | Oshana region | 50 - 300 mm of surface | rural | dry |
| **4** | Calueque-Oshakati canal | Oshana region | 50 - 200 mm of surface | rural | water |
| **5** | Oshana | Omusati region | 50 - 300 mm of surface | rural | water |
| **7** | Oshana | Omusati region | 50 - 300 mm of surface | rural | water |
| **10** | Calueque-Oshakati canal | Omusati region | 50 - 200 mm of surface | rural | water |
| **11** | Oshana | Omusati region | 50 - 300 mm of surface | rural | water |
| **12** | Oshana | Oshana region | 50 - 300 mm of surface | rural | water |
| **13** | Oshana | Omusati region | 50 - 300 mm of surface | rural | dry |
| **14** | Oshana | Omusati region | 50 - 300 mm of surface | rural | water |
| **15** | Oshana | Ohangwena region | 50 - 300 mm of surface | rural | water |
| **16** | Oshana | Omusati region | 50 - 300 mm of surface | rural | dry |
| **17** | Oshana | Omusati region | 50 - 300 mm of surface | rural | dry |
| **18** | Oshana | Omusati region | 50 - 300 mm of surface | rural | dry |
| **22** | Oshana | Omusati region | 50 - 300 mm of surface | rural | water |
| **23** | Oshana | Omusati region | 50 - 300 mm of surface | rural | water |
| **27** | Oshana | Oshana region | 50 - 300 mm of surface | urban | water |
| **29** | Oshana | Oshana region | 50 - 300 mm of surface | rural | water |
| **30** | Oshana | Oshana region | 50 - 300 mm of surface | rural | dry |
| **31** | Oshana | Oshana region | 50 - 300 mm of surface | rural | dry |
| **WO** | Oshakati | Oshana region | 50 - 200 mm of surface | urban | dry |

**Water and sediment pollution of intensively used surface waters during a drought period – a case study in Central Northern Namibia**

*Environmental Monitoring and Assessment*

Leona Faulstich, Robert Arendt, Christian Reinhardt-Imjela, Achim Schulte, Joachim Lengricht, Petrina Johannes

Freie Universität Berlin, leona.faulstich@fu-berlin.de

**Supplementary data 3**

Further parameters of the water samples of the Iishana and the Calueque-Oshakati canal (mean ± standard deviation).

|  | **Iishana** | | | **Calueque-Oshakati canal** | | |
| --- | --- | --- | --- | --- | --- | --- |
| **water** | **2017** | **2018** | **2019** | **2017** | **2018** | **2019** |
| **TNb [mg/l]** | 7.77 ± 4.58 | 6.39 ± 3.05 | 5.81 ± 3.31 | 2.07 ± 0.86 | 1.85 ± 0.69 | 1.56 ± 0.39 |
| **TC [mg/l]** | 74.99 ± 18.33 | 56.49 ± 20.08 | 81.87 ± 57.78 | 23.23 ± 0.51 | 46.86 ± 0.34 | 17.74 ± 1.29 |
| **TIC [mg/l]** | 37.92 ± 25.66 | 28.91 ± 21.49 | 44.92 ± 39.49 | 12.26 ± 2.87 | 24.80 ± 23.98 | 12.46 ± 0.76 |
| **TOC [mg/l]** | 36.90 ± 25.85 | 26.42 ± 21.33 | 36.96 ± 27.99 | 11.04 ± 2.64 | 21.95 ± 24.94 | 5.27 ± 0.86 |
| **Eh [mV]** | 217.85 ± 33.79 | 233.71 ± 28.36 | 938.78 ± 261.91 | 237.77 ± 36.95 | 236.44 ± 18.81 | 981.94 ± 517.74 |
| **dissolved O_2_ [mg/l]** | 7.8 ± 2.4 | 5.3 ± 2.7 | 9.5 ± 1.8 | 8.4 ± 0.8 | 6.9 ± 0.1 | 7.4 ± 2.6 |
| **dissolved O_2_ [sat %]** | 94.58 ± 34.36 | 89.66 ± 19.95 | 120.35 ± 25.35 | 100.83 ± 11.26 | 83.68 ± 2.69 | 103.61 ± 7.45 |
| **chlorophyll-α [µg/l]** | 40.5 ± 20.2 | 34.6 ± 14.7 | 31.4 ± 23.2 | 2.6 ± 0.7 | 13.5 ± 3.8 | 10.9 ± 2.7 |
| **cyanobacteria / BGA [cells/ml]** | // | 7879.2 ± 4395.8 | 24386.5 ± 40974.9 | // | 2063.1 ± 690.9 | 2152.3 ± 591.0 |

// = not measured

Heavy metal concentrations (in µg/l) of the water samples of the Iishana and the Calueque-Oshakati canal (mean ± standard deviation).

|  | **Iishana** | | | **Calueque-Oshakati canal** | | |
| --- | --- | --- | --- | --- | --- | --- |
| **water [µg/l]** | **2017** | **2018** | **2019** | **2017** | **2018** | **2019** |
| **As** | // | 17.60 ± 16.34 | 0.03 ± 0.04 | // | 3.60 ± 0.97 | 0.007 ± 0.003 |
| **Cd** | 0.44 ± 0.11 | 0.50 ± 0.19 | < 0.001 | 0.40 ± 0.13 | 0.29 ± 0.09 | < 0.001 |
| **Co** | 4.05 ± 3.13 | 6.86 ± 6.05 | 2.46 ± 1.38 | 2.82 ± 6.23 | 1.01 ± 1.10 | 0.33 ± 0.20 |
| **Cr** | 2.95 ± 2.67 | 3.94 ± 2.44 | 1.26 ± 0.53 | 1.73 ± 2.88 | 1.46 ± 0.59 | 0.86 ± 0.35 |
| **Cu** | 23.04 ± 35.70 | 7.00 ± 2.68 | 37.33 ± 17.19 | 44.67 ± 57.36 | 7.00 ± 5.11 | < 0.001 |
| **Ni** | 10.48 ± 10.45 | 17.70 ± 16.77 | 4.37 ± 3.53 | 7.33 ± 11.22 | 3.61 ± 1.63 | 0.98 ± 0.68 |
| **Pb** | 2.51 ± 1.27 | 2.41 ± 2.52 | 2.12 ± 1.20 | 1.88 ± 0.92 | 1.59 ± 1.19 | < 0.001 |
| **Sr** | // | 479.23 ± 285.03 | 442.84 ± 232.40 | // | 92.00 ± 54.45 | 52.90 ± 6.50 |
| **Zn** | 6.55 ± 5.28 | 16.66 ± 7.52 | 5.11 ± 4.39 | 16.51 ± 8.54 | 13.38 ± 9.69 | 3.01 ± 3.34 |

// = not measured

Heavy metal concentrations (in µg/g) of the suspended solids of the Iishana (mean ± standard deviation).

|  | **Iishana** |  |  |
| --- | --- | --- | --- |
| **suspended solids [µg/g]** | **2017** | **2018** | **2019** |
| **As** | // | 0.09 ± 0.03 | 0.17 ± 0.02 |
| **Cd** | 0.11 ± 0.02 | 0.06 ± 0.03 | 4.88 ± 6.84 |
| **Co** | 11.62 ± 1.09 | 10.30 ± 2.87 | 14.15 ± 1.15 |
| **Cr** | 63.82 ± 8.45 | 50.44 ± 16.96 | 83.72 ± 14.65 |
| **Cu** | 37.98 ± 10.91 | 30.07 ± 8.09 | 166.85 ± 130.95 |
| **Ni** | 41.26 ± 10.62 | 27.97 ± 7.10 | 52.64 ± 17.73 |
| **Pb** | 9.32 ± 2.03 | 7.02 ± 1.33 | 82.74 ± 81.57 |
| **Sr** | // | 52.97 ± 15.93 | 90.16 ± 32.85 |
| **Zn** | 111.53 ± 44.61 | 46.81 ± 6.61 | 122.79 ± 41.61 |

// = not measured

**Water and sediment pollution of intensively used surface waters during a drought period – a case study in Central Northern Namibia**

*Environmental Monitoring and Assessment*

Leona Faulstich, Robert Arendt, Christian Reinhardt-Imjela, Achim Schulte, Joachim Lengricht, Petrina Johannes

Freie Universität Berlin, leona.faulstich@fu-berlin.de

**Supplementary data 4**

Results (p-values) for significant differences in the water samples of the Iishana and the Calueque-Oshakati canal between dry and rainy seasons (df = 2).

|  | **2017 - 2018** | | | **2017 - 2019** | | | **2018 - 2019** | |  |
| --- | --- | --- | --- | --- | --- | --- | --- | --- | --- |
|  | **Iishana** | **Canal** | **Iishana** | | **Canal** | **Iishana** | | **Canal** | |
| **Al** | 0.005 | 0.018 | 0.005 | | 0.018 | 0.005 | | 0.018 | |
| **Ca^2+^** | 0.042 | n.s. | 0.042 | | n.s. | n.s. | | 0.012 | |
| **Pb** | 0.309 | 0.010 | 0.309 | | 0.010 | 0.309 | | 0.010 | |
| **Mn** | 0.847 | 0.368 | 0.847 | | 0.368 | 0.847 | | 0.368 | |
| **Fe^2+^** | 0.030 | 0.156 | 0.030 | | 0.156 | 0.030 | | 0.156 | |
| **Na^+^** | 0.513 | n.s. | 0.513 | | 0.018 | 0.513 | | n.s. | |
| **K^+^** | 0.607 | n.s. | 0.607 | | 0.004 | 0.607 | | 0.004 | |
| **Mg^2+^** | 0.311 | n.s. | 0.311 | | 0.005 | 0.311 | | 0.005 | |
| **temp** | 0.042 | 0.102 | 0.042 | | 0.102 | n.s. | | 0.102 | |
| **EC** | 0.847 | 0.156 | 0.847 | | 0.156 | 0.847 | | 0.156 | |
| **pH** | 0.115 | n.s. | 0.115 | | 0.066 | 0.115 | | n.s. | |
| **turbidity** | 0.513 | 0.005 | 0.513 | | 0.005 | 0.513 | | 0.005 | |
| **NH_4_^+^** | 0.016 | n.s. | n.s. | | n.s. | n.s. | | 0.004 | |
| **Cl^-^** | 0.311 | 0.018 | 0.311 | | 0.018 | 0.311 | | 0.018 | |
| **NO_3_^-^** | 0.011 | 0.001 | 0.011 | | n.s. | n.s. | | n.s. | |
| **NO_2_^-^** | 0.115 | n.s. | 0.115 | | n.s. | 0.115 | | 0.004 | |
| **PO_4_^3-^** | 0.115 | n.s. | 0.115 | | n.s. | 0.115 | | 0.004 | |
| **SO_4_^2-^** | 0.847 | 0.066 | 0.847 | | 0.066 | 0.847 | | 0.066 | |
| **F^-^** | n.s. | 0.002 | 0.015 | | n.s. | 0.015 | | 0.002 | |

* n.s. = not significant

Results (p-values) for significant differences in the water samples between Iishana and Calueque-Oshakati canal.

|  | **2017** | **2018** | **2019** |
| --- | --- | --- | --- |
| **Al** | 0.0106 | 0.0130 | 0.6532 |
| **Ca^2+^** | 0.0236 | 0.0034 | 0.0079 |
| **Pb** | 0.0603 | 0.1078 | 0.0281 |
| **Mn** | 0.0195 | 0.0027 | 0.4807 |
| **Fe^2+^** | 0.0749 | 0.2010 | 0.0079 |
| **Na^+^** | 0.0106 | 0.0000 | 0.0079 |
| **K^+^** | 0.0131 | 0.0001 | 0.0032 |
| **Mg^2+^** | 0.0160 | 0.0003 | 0.0001 |
| **temp** | 1.0000 | 0.0166 | 0.5317 |
| **EC** | 0.0000 | 0.0001 | 0.0006 |
| **pH** | 0.5675 | 0.6821 | 0.0079 |
| **turbidity** | 0.0000 | 0.0158 | 0.2359 |
| **NH_4_^+^** | 0.0005 | 0.1227 | 0.8093 |
| **Cl^-^** | 0.0000 | 0.0003 | 0.0001 |
| **NO_3_^-^** | 0.0057 | 0.0472 | 0.0592 |
| **NO_2_^-^** | 0.0018 | 0.0889 | 0.0076 |
| **PO_4_^3-^** | 0.0009 | 0.0153 | 0.0233 |
| **SO_4_^2-^** | 0.0025 | 0.0016 | 0.0013 |
| **F^-^** | 0.0445 | 0.4208 | 0.0093 |

Results (p-values) for significant differences in the suspended solids of the Iishana between 2017 and 2018.

|  | **2017 - 2018** |
| --- | --- |
| **Al** | 0.075 |
| **Ca^2+^** | 0.003 |
| **Pb** | 0.271 |
| **Mn** | 0.639 |
| **Fe^2+^** | 0.040 |
| **Na^+^** | 0.007 |
| **K^+^** | 0.620 |
| **Mg^2+^** | 0.693 |
